# Knowledge, attitudes and practices towards yaws and yaws-like skin disease in Ghana

**DOI:** 10.1371/journal.pntd.0005820

**Published:** 2017-07-31

**Authors:** Michael Marks, Cynthia Kwakye-Maclean, Rachel Doherty, Paul Adwere, Abdul Aziz Abdulai, Fredrick Duah, Sally-Ann Ohene, Oriol Mitja, Blanche Oguti, Anthony W. Solomon, David C. W. Mabey, Yaw Adu-Sarkodie, Kingsley Asiedu, Mercy M. Ackumey

**Affiliations:** 1 Clinical Research Department, Faculty of Infectious and Tropical Diseases, London School of Hygiene & Tropical Medicine, London, United Kingdom; 2 Hospital for Tropical Diseases, London, United Kingdom; 3 Ghana Health Services, Accra, Ghana; 4 University College London Medical School, Gower Street, London, United Kingdom; 5 World Health Organization Country Office, Accra, Ghana; 6 Barcelona Institute for Global Health, Barcelona Centre for International Health Research, Hospital Clinic, University of Barcelona, Barcelona, Spain; 7 Department of Control of Neglected Tropical Diseases, World Health Organization, Geneva, Switzerland; 8 Kwame Nkrumah University of Science and Technology, Kumasi, Ghana; 9 School of Public Health, College of Health Sciences, University of Ghana, Legon, Ghana; The Gertner Institute for Epidemiology and Health Policy Research, ISRAEL

## Abstract

**Introduction:**

Yaws is endemic in Ghana. The World Health Organization (WHO) has launched a new global eradication campaign based on total community mass treatment with azithromycin. Achieving high coverage of mass treatment will be fundamental to the success of this new strategy; coverage is dependent, in part, on appropriate community mobilisation. An understanding of community knowledge, attitudes and practices related to yaws in Ghana and other endemic countries will be vital in designing effective community engagement strategies.

**Methods:**

A verbally administered questionnaire was administered to residents in 3 districts in the Eastern region of Ghana where a randomised trial on the treatment of yaws was being conducted. The questionnaire combined both quantitative and qualitative questions covering perceptions of the cause and mechanisms of transmission of yaws-like lesions, the providers from which individuals would seek healthcare for yaws-like lesions, and what factors were important in reaching decisions on where to seek care. Chi-square tests and logistic regression were used to assess relationships between reported knowledge, attitudes and practices, and demographic variables. Thematic analysis of qualitative data was used to identify common themes.

**Results:**

A total of 1,162 individuals participated. The majority of individuals (n = 895, 77%) reported that “germs” were the cause of yaws lesions. Overall 13% (n = 161) of respondents believed that the disease was caused by supernatural forces. Participants frequently mentioned lack of personal hygiene, irregular and inefficient bathing, and washing with dirty water as fundamental to both the cause and the prevention of yaws. A majority of individuals reported that they would want to take an antibiotic to prevent the development of yaws if they were asymptomatic (n = 689, 61.2%), but a substantial minority reported they would not want to do so. A majority of individuals (n = 839, 72.7%) reported that if they had a yaws-like skin lesion they would seek care from a doctor or nurse. Both direct and indirect costs of treatment were reported as key factors affecting where participants reported they would seek care.

**Discussion:**

This is the first study that has explored community knowledge, attitudes and practices in relation to yaws in any endemic population. The belief that ‘germs’ are in some way related to disease through a variety of transmission routes including both contact and dirty water are similar to those reported for other skin diseases in Ghana. The prominent role of private healthcare providers is an important finding of this study and suggests engagement with this sector will be important in yaws eradication efforts. Strategies to address the substantial minority of individuals who reported they would not take treatment for yaws if they were currently asymptomatic will be needed to ensure the success of yaws eradication efforts. The data collected will be of value to the Ghana Health Service and also to WHO and other partners, who are currently developing community mobilisation tools to support yaws eradication efforts worldwide.

## Author summary

Yaws, a bacterial skin infection, is endemic in Ghana. WHO has launched a campaign to eradicate yaws based on community mass treatment with the antibiotic azithromycin. Community perceptions of disease are an important contributor to participation in mass treatment interventions. This study used questionnaires to understand beliefs about yaws amongst individuals living in endemic communities in Ghana. Most individuals reported that ‘germs’ were the cause of yaws although the route of transmission was less well understood with many individuals reporting that dirty drinking or washing water was responsible for transmission. Participants reported they would normally seek care within the formal healthcare sector although many individuals reported they would visit traditional healthcare providers or pharmacies. Cost of care was the key factor for many participants. A majority of individuals reported they would be happy to take an antibiotic to prevent infection but a large minority (38.8%) reported that they would not. This study provides the first data on community beliefs about yaws in Ghana and will be valuable in helping the Ghana Health Service and partners develop community mobilisation tools to support yaws eradication efforts worldwide.

## Introduction

Yaws, caused by *Treponema pallidum* subsp. *pertenue*, is endemic in Ghana. The disease is reported in all districts but predominantly in the south of the country. The majority of clinical disease is seen in young children, with the organism being transmitted by skin to skin contact with infectious lesions. In 2012, the World Health Organization (WHO) launched a new global eradication campaign based on total community mass treatment (analogous to mass drug administration—MDA) with azithromycin [1]. This involves the treatment of all individuals in an endemic community regardless of the presence or absence of clinical yaws. Achieving high coverage of mass treatment will be fundamental to the success of this new strategy [2]; coverage is dependent, in part, on appropriate community mobilisation. The success of interventions may be jeopardized by non-participation within endemic communities [3], because it may leave reservoirs of infection from which disease may re-emerge.

Studies in Ghana have established that amongst residents of rural communities there is a strong conceptual connection between lack of cleanliness and disease causation [4–6]. In a study conducted within Bono (Akan) society, sickness and disease may be attributed to many causes, including water (*nsuo*), dirt (*ef*i,) lack of personal hygiene, cleanliness, the environment in which one sleeps, and bad air or wind (*mframa*) [6]. Another study reported that beliefs about dirty bodies (*shoefi*) are central to the understanding of disease for the Akan, contributing to the importance attached to daily bathing [5]. Many traditional belief systems incorporate a strong correlation between microorganisms (*mmoa*) and dirt (*efi*), in which efi is “synonymous with contamination and disease, and implies the necessity of cleansing and purification” [6]. Community perceptions of disease causation may play an important role in access to or utilization of health services [7,8].

There are no publications on community knowledge, attitudes and practices (KAP) related to yaws in Ghana or other endemic countries. However, an understanding of these issues will be vital in designing effective community engagement strategies to support the yaws eradication effort. This study was conducted alongside a randomised control trial investigating the optimal dose of azithromycin for treatment of yaws in Ghana. The study was conducted in three districts (total population 319,898) which had reported 1,505 cases of yaws in the preceding 36 months, and aimed to collect both quantitative and qualitative data on local knowledge, attitudes and practices concerning the causation and treatment of yaws.

## Methods

### Questionnaire development and trial

A verbally administered questionnaire was developed, based on existing information about yaws and previous studies on community beliefs around skin disease in West Africa [9,10]. Prior to commencing this study, the questionnaire was pre-tested with a small number of participants from a group of communities not included in recruitment of the definitive sample of respondents. The questionnaire was refined based on this experience to ensure clarity of questioning.

### Study setting and participant recruitment

This study was conducted in 3 districts in the Eastern region of Ghana namely Ayensuanor, West Akyem and Upper West Akyem in 2015. These contiguous districts were selected out of 4 districts where a randomised trial on different doses of azithromycin for the treatment of yaws was being conducted (ClinicalTrials.gov identifier NCT02344628). The fourth district was 400 km away and could not be included in the KAP study because of distance and costs reasons. None of these districts had previously received azithromycin mass treatment for yaws or trachoma. Within the chosen districts, multi-stage sampling was used to select participants for the adjunctive investigations described here. The first level of sampling was communities in the study districts which were selected using simple random sampling. In each selected community, an estimate of the total adult population was obtained from the district directorate of health services. In selected communities, systematic sampling was used to select participating households by randomly selecting an initial house and then utilising a quasi-random clockwise walking method [11,12]. In each house, either the oldest male or female resident was interviewed. If no adults were present at the time of visit, the house was excluded. In order to obtain a gender-balanced survey, the selection of respondent was alternated by gender between houses where possible.

### Questionnaire administration

Questionnaires were administered by local staff from each district. Participants were shown photos of typical yaws lesions as examples of the disease being discussed. Data were collected on demographics, including gender, ethnicity, religion, age, highest level of education achieved and occupation. The questionnaire combined both quantitative and qualitative questions covering several themes, including perceptions of the cause and mechanisms of transmission of yaws-like lesions, the providers from which individuals would seek healthcare if they themselves had a yaws-like lesions, and what factors were important in reaching decisions on where to seek care. Prior to conducting fieldwork, all staff received training on qualitative techniques and on administering the questionnaire, with training provided by the lead member of the KAP study team (MMA). Questionnaires were administered in local languages, and recorded on a standard form. Data were entered into a study database in EpiInfo by one of two members of the study team (either RD or BO).

### Data analysis

Quantitative data were analysed with the use of descriptive statistics. Categorical variables were summarized using absolute numbers and percentages. Chi-square tests and logistic regression were used to assess relationships between reported knowledge, attitudes and practices, and demographic variables, including age, ethnicity, religion and level of education. These analyses were performed using STATA 13.1 (Statacorp). For the purposes of qualitative analysis, we considered *knowledge* to be participants’ reported beliefs around the causation and transmission of yaws, *attitudes* to reflect what individuals believed could or should be done to prevent disease, and *practices* to encompass the healthcare seeking behaviour of individuals with yaws and the underlying reasons for presenting. Qualitative data were assessed across each of these domains. Data were manually codified to identify common themes. Representative examples of major themes were identified in each domain.

### Ethics approval

The studies were approved by the World Health Organization (RPC 720), London School of Hygiene & Tropical Medicine (LSHTM 8832) and Ghana Health Service (GHS 13/11/14) ethics committees. Written informed consent was obtained from all participants.

## Results

### Background characteristics of respondents

A total of 1,162 individuals participated. Slightly more women (n = 600, 51.6%) were included than men, and the median age of participants was 36 years (IQR 27–46 years). The majority of participants were Christians (n = 966, 83.1%) and of Akan ethnicity (n = 593, 51.0%); other reported religious affiliations and ethnicities are shown in Table 1. A minority of participants reported a personal history of a lesion consistent with yaws (n = 214, 18.4%).

**Table 1:** Demographic characteristics.

		n	%
**Gender**	Male	537	46.3%
	Female	600	51.7%
	Missing Data*	25	2.2%
**Religion**	Christian	966	83.2%
	Muslim	100	8.7%
	Traditionalist	17	1.5%
	None	39	3.4%
	Missing Data*	40	3.5%
**Ethnicity**	Akan	593	51.1%
	Ewe	200	17.3%
	Ga-Abangbe	124	10.7%
	Northern Ethnic Tribe	85	7.4%
	Non-Ghanaian	3	0.3%
	Missing Data*	157	13.6%
**Marital Status**	Single	286	24.7%
	Widowed	81	7%
	Divorced/Separated	89	7.7%
	Married	680	58.6%
	Missing Data*	26	2.3%
**Occupation**	None/ No formal employment	499	43.1%
	Semi-Skilled Work	615	53%
	Skilled Work	28	2.5%
	Professional	20	1.8%
	Missing Data*	71	6.2%
**Education**	None	241	21.0%
	Primary (Approximately age 6–12)	292	25.2%
	Junior High School (Approximately age 13–15)	487	42%
	Senior High School (Approximately age 16–18)	120	10.4%
	Tertiary Education	17	1.5%
	Missing Data*	15	1.3%

*Incomplete or illegible information recorded for this question

### Knowledge: Local perceptions of causation

The majority of individuals (n = 895, 77%) reported that “germs” were the cause of yaws lesions. Contact with an individual with a similar lesion was reported to be an important cause by 532 participants (45.8%). Many individuals (n = 816, 70.2%) incorrectly believed that washing in or drinking dirty water were possible routes of transmission (Table 2). There was substantial overlap in beliefs with the majority of those who believed contact with an infected individual was a route of transmission also believing that washing or drinking water played a role (n = 457, 85.9%). In open-ended questions many respondents (38.1%) also suggested transmission was more broadly related to hygiene, contact with dirty water or a lack of washing. 13%(n = 161) of respondents believed that the disease was caused by supernatural forces such as witchcraft, curses or a punishment from god. Belief in a supernatural cause was not associated with gender, religion or ethnic group (p > 0.05 for all comparisons).

**Table 2:** Knowledge, attitudes and practices towards yaws-like lesions^.

Domain	n*	%*
**Reported causes of yaws-like lesions**		
Germs	895	77.1%
Contact	532	45.8%
Washing with Dirty Water	610	52.5%
Drinking with Dirty Water	590	50.8%
Insect Bites	224	19.3%
Witchcraft	129	11.2%
Curse	83	7.2%
Punishment from God	30	2.6%
Other**	365	31.5%
No answer given	179	15.4%
**Where would you try to seek care**		
Nurse	695	59.9%
Doctor	518	44.6%
Traditional Healer	481	41.4%
Witch Doctor	38	3.3%
Pharmacy	426	36.7%
Shop	28	2.5%
Teacher	24	2.1%
Family	57	5%
No Answer Given	3	0.3%
**Factors affecting where to seek care**		
Cost of Travel	646	55.6%
Cost of Treatment	1029	88.6%
What my family think	51	4.4%
Previous Experience	50	4.4%
Experience of other individuals	43	3.8%
No Answer Given	179	15.4%
**Treatments participants reported using**		
Gentian	434	37.3%
Topya	434	37.3%
Animal Products	36	3.1%
Dressings	224	19.3%
Tablets	505	43.5%
Injections	537	46.2%
No Answer Given	27	2.3%

*Participants could report more than one answer for some questions and therefore the total adds up to more than the total number of participants in the study

^ Answers based on closed question on possible causes

** This category included a broad range of responses related to hygiene but without specifying dirty water or contact with an unwell individual

Participants frequently mentioned lack of personal hygiene, irregular and inefficient bathing, and washing with dirty water as fundamental to both the cause and the prevention of yaws.

“*Germs from dirty things cause some skin problems*”

(27-year-old Ewe woman, Ayensuano)

“*If you don’t bathe with soap and water, you will get infected*”

(18-year-old Ewe man, Upper West Akim)

More broadly, many participants asserted that bathing and personal hygiene were closely associated to wider notions of longevity of life and health, stating that bathing was fundamental to healthy living.

“*It helps one to maintain good health and live a long life*”

(34-year-old Akan woman, Ayensuano)

“[Personal hygiene is important] *to prolong our life and be healthy”*

(38–year-old woman, northern ethnic tribe, Ayensuano)

Contact with individuals and/or sharing of sponges or other items used in bathing were also reported as playing an important role in disease transmission, while some individuals reported a belief that transmission could be caused by airborne spread of germs.

“*If you use the same sponge as the affected person you may get it*”

(24-year-old Akan woman, Ayensuano)

“*[You must] avoid close contact to anybody with rashes and any skin diseases…because the disease can spread throughout the air*”

(60-year-old Ga-Adangbe woman, Ayensuano)

### Attitudes

The over-riding theme emerging from the interviews was that dirt, dirtiness and germs were inextricably connected to notions of cleanliness in understanding the mechanism of transmission, and that personal hygiene was believed to be the key intervention to protect individuals from germs and dirt—and therefore from infection.

“*The infection occurs when one doesn’t observe good body hygiene*”

62 year old Akan man, Upper West Akim

“*If you maintain personal hygiene it helps you to live longer [free] from skin problems*”

31-year-old Ewe woman, Ayensuano

A majority of individuals reported that they would want to take an antibiotic as part of a mass treatment campaign to treat yaws, even if they themselves were asymptomatic (n = 689, 61.2%), but a substantial minority reported they would not want to do so (31.8%). After adjustment for confounders, neither religion nor level of education were associated with individuals reporting that they would accept treatment for yaws (p = 0.06 and p = 0.09 respectively). After controlling for other factors individuals of Ga-Adangbe were slightly more likely to report willingness to accept treatment (OR 1.6 95% CI 1.05–2.45, p = 0.01). After controlling for demographic factors, belief in supernatural causation of yaws-like lesions was associated with a non-significant lower likelihood of accepting medication (OR 0.53–95% CI 0.07–3.99, p = 0.541).

### Practices: Care seeking

A majority of individuals (n = 839, 72.7%) reported that if they had a yaws-like skin lesion they would seek care from a doctor or nurse. A substantial minority of individuals reported that they would seek care through the private sector, most commonly a pharmacy (n = 426, 37%) or a private shop (n = 28, 2.4%) (Table 2). Many individuals also reported that they would seek care from a traditional healer or witchdoctor (n = 496, 42.7%). Belief in a supernatural cause for yaws was strongly associated with the individual reporting that they would seek care from a traditional healer or witchdoctor (OR 2.93–95%CI 2.1–4.2, p <0.0001). Many individuals (n = 276, 36.8%) reported that they would seek care both from the formal healthcare sector (doctor, nurse, hospital) and a traditional healer or witchdoctor.

### Practices: Determinants of care seeking

Cost was the factor most commonly reported by respondents as being important in determining where they sought care (n = 1,080 92.9%), with both the direct cost of treatment (n = 1,029, n = 88.6%) and the cost of travel (n = 646, 55.6%) reported as considerations. The opinions of family members or an individual’s previous experience were relatively infrequent considerations (n = 118, 10.2%) in guiding treatment seeking behaviours (Table 2). The qualitative data confirmed that cost was a major driver in decision making about where to seek care:

“*I stay far from the hospital so I have to walk about 5 miles before I reach the hospital, so it is very difficult for me to get money to go to the hospital as well as transportation*”

(48-year-old Akan woman, Ayensuano)

“*I have to seek help from a place nearer to me to avoid the high cost*”

(43-year-old Ewe woman, Ayensuano)

### Practices: Treatments received

Participants reported receiving a wide range of different treatments for yaws-like lesions. The most commonly reported treatments were tablets (n = 505, 43.6%) or injectable antibiotics (46.2%) but many people also reported receiving topical treatments (Table 2).

## Discussion

We have explored community knowledge, attitudes and practices with regards to yaws in southern Ghana, a region of the country where yaws is endemic. Whilst most participants reported that infection was in some way related to “germs”, they reported a variety of perceived routes of transmission, including both correct (contact with infected individuals) and incorrect (e.g, contact with dirty water) routes of transmission. These findings are in keeping with studies undertaken in Ghana concerning knowledge, attitudes and practices in relation to Buruli ulcer, which have emphasised the perceived role of contact with infected individuals and drinking dirty water as important routes of transmission for that disease [9,10]. In the study reported here, almost 15% of participants believed that witchcraft or curses could be responsible for skin ulcers. Attributing an illness to supernatural forces such as witchcraft and curses is a common phenomenon and is often due to a lack of knowledge of the true aetiology of illness, particularly when the illness is prolonged [9]. Many individuals in Buruli ulcer endemic areas of Ghana report similar beliefs about causation of disease [9,10].

Whilst the majority of individuals reported that they would seek care from a doctor or a nurse for the management of yaws-like lesions, the prominent role of private healthcare providers is an important finding of this study. In Ghana, most individuals are eligible for free health care via a national health insurance programme and most individuals have access to a local primary health care facility. However indirect costs and time to travel to state providers may still represent a barrier to accessing ‘free’ health-care and may be a reason for seeking alternative care provider if that is more readily available locally [13]. As in other parts of sub-Saharan Africa private pharmacies are also common. There individuals may purchase a wide-range of medications directly without the need for medical consultation. More than a third of individuals reported that they would seek care from private providers: specifically staff at private pharmacies or traditional healers. Acceptance of a given intervention can depend on a variety of factors, including understanding of causation, perceived effectiveness of the intervention and both direct and indirect cost factors [7]. Our findings that cost and distance represent barriers to accessing healthcare are in keeping with other studies from Ghana [14,15]. Whilst community-based MDA programmes should address cost issues, health education will be needed to address beliefs around causation and the effectiveness of azithromycin against yaws. Yaws eradication programmes should also consider how best to engage with these other providers, who operate outside of the formal healthcare system, and who may be an essential means for detecting cases of yaws in endemic communities as the eradication endpoint is approached. They should be considered both in Ghana and in other settings where such informal providers play important roles alongside formal healthcare systems. As the majority of yaws cases occur amongst children, consideration could also be given to training school teachers to identify signs and symptoms of yaws, and engaging them in the yaws eradication effort and further studies exploring this possibility should be undertaken.

A sizeable minority of individuals in our study reported that they would not be willing to take medicine for yaws if they were asymptomatic at the time that it was offered to them. This is potentially worrisome, as estimates suggest that for each symptomatic case of yaws, there are 5–10 individuals with asymptomatic infection, and that latent yaws can reactivate up to a decade after primary disease has resolved in an individual; this, in fact, is part of the basis for the use of an initial strategy of total community treatment, rather than one involving identification and treatment of those with disease [16]. If our unwillingness rate was translated into non-participation rates in yaws eradication campaigns, it would represent a significant challenge for interrupting transmission in Ghana and likely increase the number of rounds of treatment required to reach that goal [2].

Studies of the acceptability of azithromycin MDA for trachoma have reported associations with gender and beliefs around disease causation [7]. In our respondents, we did not identify any clear demographic factors associated with the likelihood of treatment refusal when asymptomatic. The majority of cases of yaws occur in children (who were not included in this survey) but in the context of MDA for trachoma, the decision of the household head is often important in determining whether any members of a household receive azithromycin [3]. Interventions that increase the acceptability of treatment to household adults might also result in improved uptake of treatment amongst children.

Individuals who reported a supernatural belief about causation, such as witch-craft or a curse, were more likely to report that they would not accept treatment, but this association did not reach statistical significance. More in-depth qualitative work should be considered to explore this issue in greater detail.

This study has a number of limitations. First, we did not conduct in-depth interviews alongside the questionnaires. This may have limited our ability to explore issues arising from the questionnaire in more detail, or to fully explore the nuances of individual or community beliefs around yaws. Second, the clinical lesions of yaws are similar to many other skin diseases and we cannot be certain of the extent to which the beliefs expressed by community members are specific to yaws rather than applying more broadly to locally endemic skin diseases. Clinically important differentiations between diseases such as scabies, Buruli ulcer, yaws and leprosy may not be easily discernible to community members, although Buruli ulcer is not known to be endemic in any of the districtsin which this study was conducted. Although MDA with azithromycin may have ancillary benefits in treating some other skin diseases [17,18] it will not affect many common skin infections, such as Buruli ulcer, scabies or tinea. Health education programmes will need to clearly emphasise that whilst azithromycin will have a significant impact on yaws and some other diseases, it is not a panacea for all skin complaints. Finally we cannot be certain that individuals would always seek care for their children given how common skin disease is in many communities, nor that they would seek care for their children in the same place that they stated they would seek care for themselves. Data from other studies suggests that the attitude of the adult or head of household dictates engagement with healthcare services for all household members [3] so we believe that the viewpoints of adults would be key in determining healthcare entry points for children.

Despite these limitations, this is the first study of which we are aware that has explored community knowledge, attitudes and practices in relation to yaws in any endemic population. We were able to collect quantitative and qualitative data from a broad cross-section of individuals across a range of ages, genders, and ethnic and religious groups in three districts in Ghana. Our data highlight the need to address barriers to accessing care including both direct and indirect healthcare costs. The data collected will be of value to the Ghana Health Service and also to WHO and other partners, who are currently developing community mobilisation tools to support yaws eradication efforts worldwide.

## Supporting information

S1 File Supplementary data file. (XLS)

S2 File Example photos used in survey. (TIF)
