# Supplementary figures and images for: Knowledge, attitudes and practices towards yaws and yaws-like skin disease in Ghana
Source: PLoS Negl Trop Dis. 2017 Jul 31;11(7):e0005820. doi: 10.1371/journal.pntd.0005820 (PMC5552343; doi:10.1371/journal.pntd.0005820)

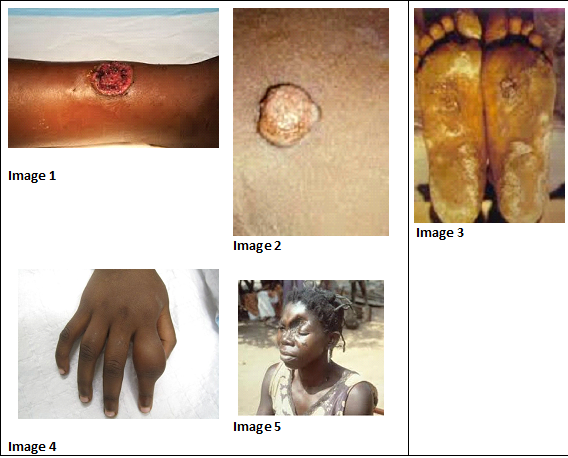

Supplement: S2 File — (TIF) [file pntd.0005820.s002.tif]
